# Effect of Pectinolytic Enzyme Pretreatment on the Clarification of Cranberry Juice by Ultrafiltration

**DOI:** 10.3390/membranes11010055

**Published:** 2021-01-14

**Authors:** Véronique Perreault, Noémie Gouin, Amélie Bérubé, William Villeneuve, Yves Pouliot, Alain Doyen

**Affiliations:** Department of Food Sciences, Institute of Nutrition and Functional Foods (INAF), Université Laval, Quebec, QC G1V 0A6, Canada; veronique.perreault.5@ulaval.ca (V.P.); noemie.gouin.1@ulaval.ca (N.G.); amelie.berube.3@ulaval.ca (A.B.); william.villeneuve.1@ulaval.ca (W.V.); yves.pouliot@fsaa.ulaval.ca (Y.P.)

**Keywords:** cranberry juice, pectinolytic enzyme, ultrafiltration, membrane molecular weight cut-off, clarification, membrane fouling, polyphenols

## Abstract

Cranberries, mainly processed as juice, have garnered interest over the past decade due to their high content of phytochemical compounds related to promising health benefits. To meet consumer expectations, a juice clarification step is usually incorporated to remove suspended solids. The use of pectinolytic enzyme and membrane processes are commonly applied to the production of clarified juices, but no studies have been done on cranberry juice. In this study, the effects of 60 (D60) and 120 min (D120) of depectinization by pectinolytic enzymes coupled to clarification by ultrafiltration (UF) (membrane molecular weight cut-off (MWCO) of 50, 100 and 500 kDa) was evaluated on the filtration performance, membrane fouling and cranberry juice composition. Compared to fresh juice, depectinization for 60 and 120 min reduced the UF duration by 16.7 and 20 min, respectively. The best filtration performance, in terms of permeate fluxes, was obtained with the 500 kDa MWCO UF membrane despite the highest total flux decline (41.5 to 57.6%). The fouling layer at the membrane surface was composed of polyphenols and anthocyanins. Compared to fresh juice, anthocyanin decreased (44% and 58% for D60 and D120, respectively) in depectinized juices whereas proanthocyanidin (PAC) content increased by 16%. In view of the industrial application, a 60 min depectinization coupled to clarification by a 500 kDa UF membrane could be viewed as a good compromise between the enhancement of filtration performance and the loss of polyphenols and their fouling at the membrane surface.

## 1. Introduction

Cranberry (*Vaccinium macrocarpon*) is a berry mostly cultivated in the United States and Canada, assuming 76% and 21% of the world production, respectively [1]. Quebec is responsible for over half of all Canadian production, and its export worth was valued at more than $124 million CAD in 2016 [2]. As with any fruit, cranberry is mainly composed of water (87.5% *w*/*w*) and carbohydrates (8.5% *w*/*w*), including dietary fiber (4.5% *w*/*w*) [1]. Moreover, due to its polyphenol content—this fruit is rich in flavonoids, including anthocyanins and proanthocyanidins (PACs) [3]—cranberry is increasingly recognized for its health benefits. Many clinical studies have indicated that polyphenols, including the categories found in cranberry, positively impact cardiovascular health, oxidative stress and inflammation [4,5,6]. Recent studies have shown the antimicrobial action of cranberry PACs on different microorganisms involved in the prevention of urinary tract infections, stomach ulcers and cancers, and the promotion of buccal health, including *Escherichia coli*, *Helicobacter pylori*, *Candida albicans* and *Pseudomonas aeruginosa* [5,6,7,8].

Cranberry must be processed to encourage its consumption since the fresh fruit has tart and astringent tastes. One of its most popular forms is cranberry juice. The province of Quebec processes over 95% of its harvest and cranberry juice makes up more than 25% of the finished products [2]. Since fresh juices contain suspended solids that precipitate, one of the critical steps to achieve a marketable product prior to filtration is clarification [9]. The use of a clarifying agent, such as gelatin, bentonite or silica sol, is one of the most common methods to achieve this [10]. Chitosan has also been used for the clarification of apple, grape and orange juice [11]. Based on electrostatic interactions, the principle is to form insoluble flocs between floating particles and clarifying agents [12]. The main disadvantages of using clarifying agents are the production of a large amount of waste and changing the polyphenolic composition of the juice [10,13].

Pressure-driven membrane processes such as ultrafiltration (UF) are widely used for the clarification of fruit juices, mainly for the removal of pectin residues [14]. The UF-membranes used for juice treatment have a molecular weight cut-off (MWCO) between 10 and 500 kDa, corresponding to the wide range of pectin molecular weight values (10–500 kDa) [15]. The permeate recovered from UF of fresh or pasteurized juice is considered to be clarified juice, since insoluble components (fiber, pectin, cellulose, etc.) do not pass through the membrane and are concentrated in the retentate. Ultrafiltration (UF) has many benefits compared to traditional methods as it combines clarification and filtration in one step, without the use of clarifying and fining agents [16]. Moreover, many studies have demonstrated that the use of filtration membranes to clarify juices decreases turbidity by removing suspended solids without affecting composition, polyphenol content or conservation [16,17,18]. However, membrane fouling is the main limiting factor of membrane processes. It drastically decreases the permeate flux and the membrane’s useful lifetime, resulting in significant productivity loss and increased production costs and energy requirements [19].

Several compounds, such as proteins, pectin and polyphenols, are known to contribute to membrane fouling during juice filtration [20,21]. The use of pectinolytic enzymes for the depectinization step has been effective in reducing the impact of membrane fouling [22]. Indeed, different enzymes, such as pectinase or cellulase, are available at commercial-scale and are generally added to the fresh juice prior to filtration [23]. During depectinization, high molecular weight compounds are cleaved to facilitate filtration by minimizing their deposition at the membrane surface while also decreasing the juice viscosity [19]. Depectinization enhances the performance of membrane processes during juice filtration since permeate fluxes are improved, as has been demonstrated for lemon and apple juices [24,25]. Optimal operational conditions for depectinization duration and UF parameters are well-documented for a large number of fruit juices but there are no studies on the clarification of cranberry juice by UF. Consequently, this study evaluates the efficacy of depectinization by pectinolytic enzymes as a pretreatment prior to clarification by UF membrane on the performance of the UF system and the cranberry juice composition. More specifically, the objectives of this study were (1) to evaluate the impact of the depectinization process for different membrane molecular weight cut-offs (MWCO) on the performance of cranberry juice UF, (2) to assess the influence of the process conditions on the composition and physicochemical characteristics of cranberry juice and (3) to characterize the main fouling components at the membrane surface.

## 2. Materials and Methods

### 2.1. Cranberry Juice and Depectinization

Fresh (non-pasteurized) cranberry juice, separated into three batches, was kindly provided by Fruit D’Or (Plessisville, QC, Can). The three batches were slowly centrifuged with a basket centrifuge using a 400 µm filter (CEPA/TZ 5, Sysbiotech, Perigny, France) at approximately 1000 rpm to remove suspended solids. The first batch was then frozen at −20 °C, while the other two were depectinized prior to storage at −20 °C. Depectinization was performed with a pectinase (Klerzyme^®^ 150, Centerchem, Norwalk, CT, USA) at a concentration of 0.04% *v/v* at 50 °C for 60 min (D60) or 120 min (D120). The enzyme was inactivated by heating the juice at 80 °C for 2 min [26]. Fresh (F) and depectinized juices (D) were frozen in 1 L containers and thawed at 4 °C one day prior to filtration or depectinization experiments.

### 2.2. Ultrafiltration System

UF of fresh and depectinized juices was performed in a crossflow filtration unit (SEPA-CF, Sterlitech, Kent, WA, USA) as described by Leu et al. [27]. A refrigeration coil maintained the fresh and depectinized juices at a constant temperature of 15 °C. The membrane module consisted of a polyvinylidene difluoride (PVDF) flat-sheet UF membrane (Synder filtration inc. Vacaville, CA, USA) with an active filtration surface of 0.014 m^2^ and spacer thickness of 31 mil on the feed side. Three different membrane MWCO were tested: 50, 100 and 500 kDa (models BN, BY, A6, respectively). Membranes were conditioned with a chlorinated alkaline solution (pH 10.5, 50 °C, 20 min, <200 mg/kg of free chlorine) and rinsed with distilled water until neutral pH was reached. Prior to juice UF, pure water flux was measured in duplicate and only UF membranes with similar water flux (±15%) were selected for use in this study.

### 2.3. Operational Modes

#### 2.3.1. Total Recycle Mode

Total recycle experiments were carried out at 15 °C and different transmembrane pressures (TMP) to determine the optimal operational conditions for the concentration mode. For total recycle experiments, retentate and permeate of fresh and depectinized juices were recycled into the feed tank. The permeate flux was measured at TMPs from 112 to 650 kPa depending on the juice (fresh or depectinized) at a constant retentate recirculation rate of 360 kg·h^−1^ and crossflow velocity of 0.62 m·s^−1^. Each pressure level was maintained for 10 min until a stable flux value was reached. The TMP for each juice was calculated according to Equation (1) [28]:

(1) $$\mathrm{TMP}=\frac{P_{i}+P_{o}}{2}$$

where P_i_ and P_o_ are the pressure (kPa) at the inlet and the outlet of the membrane cell.

Permeate flux J (g/m^2^·h) was calculated according Equation (2):

(2) $$J=\frac{m_{p}}{t\cdot A}$$

where *m_p_* is the weight of the permeate (g), *t* is the time (h) and *A* is the total filtration surface area (m^2^).

At the end of the recycle mode experiments, critical flux (J_cri_t) was defined as the lowest flux that creates an irreversible deposit on the membrane surface [29] and limiting flux (J_lim_) was defined as the maximum stationary permeate flux that can be reached when increasing TMP [30]. J_crit_ and J_lim_ were determined for fresh and depectinized juices ultrafiltered with membrane MWCO of 50, 100 and 500 kDa.

#### 2.3.2. Concentration Mode

Concentration experiments of fresh and depectinized juices were performed at 15 °C in batch mode, with simultaneous retentate recycling and permeate removal at the optimal TMP obtained from total recycle mode experiments and corresponding to 80% of the critical flux (J_crit_). Juices were concentrated until reaching a volume concentration factor (VCF) of 3X. Permeate flux was measured every 10 min. At the end of the filtration, permeate (clarified juice) and retentate were collected and frozen until the determination of proximate composition. Ultrafiltration membranes were stored at 4 °C until fouling layer extraction and characterization. UF was performed in triplicate for each depectinization treatment and UF membrane MWCO.

### 2.4. Physicochemical Analysis of Fresh and Depectinized Juices and Ultrafiltration Permeates

Suspended solids (°Brix) were measured with a refractometer (PAL-1, Atago, Tokyo, Japan) having a limit of detection in the range 0.0–53.0% *w*/*w*. Clarification of cranberry juices before and after UF treatments were determined by transmittance measurement on a spectrophotometer at 660 nm (Helios Alpha, Thermo Spectronic, Alva, United Kingdom). The pH was recorded using a handled pH-meter (VWR Symphony pH-meter model SP20, Thermo Orion, West Chester, PA, USA). The titratable acidity (TA) of juices was measured according to AOAC method 942.15. Briefly, 10 mL of sample was diluted in 40 mL of degassed distilled water. NaOH 0.1 N was added until the pH reached 8.2 [31]. The results were expressed in g/L of citric acid monohydrate equivalent.

### 2.5. Analysis of Phenolic Fractions of Fresh and Depectinized Juices and Ultrafiltration Permeates

#### 2.5.1. Total Phenolic Content

Total phenolic content was determined by using the Folin–Ciocalteu assay described by Singleton and Rossi [32]. Briefly, samples of fresh and depectinized juice (D60 and D120) as well as their respective UF permeates were diluted by a factor of 2. A volume of 20 µL of each sample was added to 100 µL of Folin–Ciolcalteu’s reagent previously diluted at 1/10. Four minutes later, 80 µL of 7.5% Na_2_CO_3_ was added to inactivate the reagent. Gallic acid standards of 50, 100, 250 and 500 mg/mL mg/L were prepared (Sigma Aldrich, Saint-Louis, MO, USA) to generate a calibration curve. These concentrations were chosen since they represent the polyphenol content in cranberry juice. The microplate was incubated at room temperature in the microplate reader before reading the absorbance at 765 nm with a xMark Microplate spectrophotometer (Bio-Rad, Mississauga, ON, Can). The results were expressed in mg/L of gallic acid equivalent.

#### 2.5.2. Anthocyanin Content

Individual anthocyanin content was measured on fresh, depectinized and permeate samples by high-performance liquid chromatography (HPLC) with an Agilent 1100 serie with a UV detector (Agilent, Santa Clara, CA, USA). A volume of 800 µL of fresh juice, depectinized juice (D60 and D120), or permeates of fresh and depectinized juices (D60 and D120) was added to 200 µL of methanol containing 0.5% of trifluoroacetic acid and were filtered on a 0.45µm nylon filter. A small volume (20 µL) was then injected onto a ZORBAX SB-C18 5µm (4.6 × 250 mm column (Agilent, Santa Clara, CA, USA) at room temperature with 2 mobile phases (A: 5% formic acid solution and B: methanol 100%) at a flow rate of 1 mL/min. The column was eluted over 90 min and detection was done at 520 nm. Standard anthocyanins (cyanidin-3-glucoside) was used to identify the unknown peaks in cranberry juice samples. The results were expressed in ppm of cyanidin-3-glucoside equivalent. Data were recorded and analyzed with the ChemStation Rev. C. 01.07 SR1 software (Agilent, Santa Clara, CA, USA).

#### 2.5.3. Proanthocyanidin Content

Proanthocyanidins (PACs) were measured by HPLC with an Agilent 1260 (Agilent, Santa Clara, CA, USA) equipped with a fluorescence detector. Briefly, 295 µL of fresh juice, depectinized juice (D60 and D120), or permeates of fresh and depectinized juices (D60 and D120) were added to 705 µL of acetone containing 0.71% glacial acetic acid. This solution was then filtered on a 0.45 µm nylon filter and 5 µL was injected onto a Nomura Chemical Develosil 100 Diol-5 (4.6 × 250 mm, Phenomenex column, Torrance, CA, USA) at 35 °C. Mobile phase A (ACN: acetic acid 98%/2%) and mobile phase B (95% methanol/3% water/2% acetic acid) were used at a flow rate of 0.8 mL/min. PACs detection was performed at 321 nm after excitation at 230 nm. Standard proanthocyanidins (epicatechin) was used to identify the unknown peaks in cranberry juice samples. The results were expressed in ppm of epicatechin equivalent. Data were recorded and analyzed with the ChemStation Rev. B. 04. 03-SP1 software (Agilent, Santa Clara, CA, USA).

### 2.6. Membrane Fouling Deposit Analysis

After UF of fresh and depectinized juices in concentration mode, UF membranes were soaked in a 60%/40% (*v*/*v*) acetone/water solution for 3 h for foulant extraction. Acetone was completely evaporated in a rotatory evaporator (Rotavapor R-215, Büchi, Flawil, Switzerland). Following the extraction, no apparent trace of the fouling was visible at the surface of UF membranes. The same protocols described for fresh and depectinized juices as well as UF permeates were followed for the determination of total phenolic compounds, anthocyanins and PACs from solutions recovered from the UF membrane surface after extraction experiments.

### 2.7. Statistical Analysis

Treatments followed a 3 × 3 factorial structure (MWCO × depectinization). UF experiments and analyses were performed in triplicate. A two-way analysis of variance (ANOVA) was performed at a 95% confidence level. Fisher’s protected least significant difference (LSD) was used to compare membranes cut-off and juice depectinization treatments (SAS software, version 9.4, SAS Institute Inc., Cary, NC, USA).

## 3. Results

### 3.1. Total Recycle Mode

**Figure 1:**
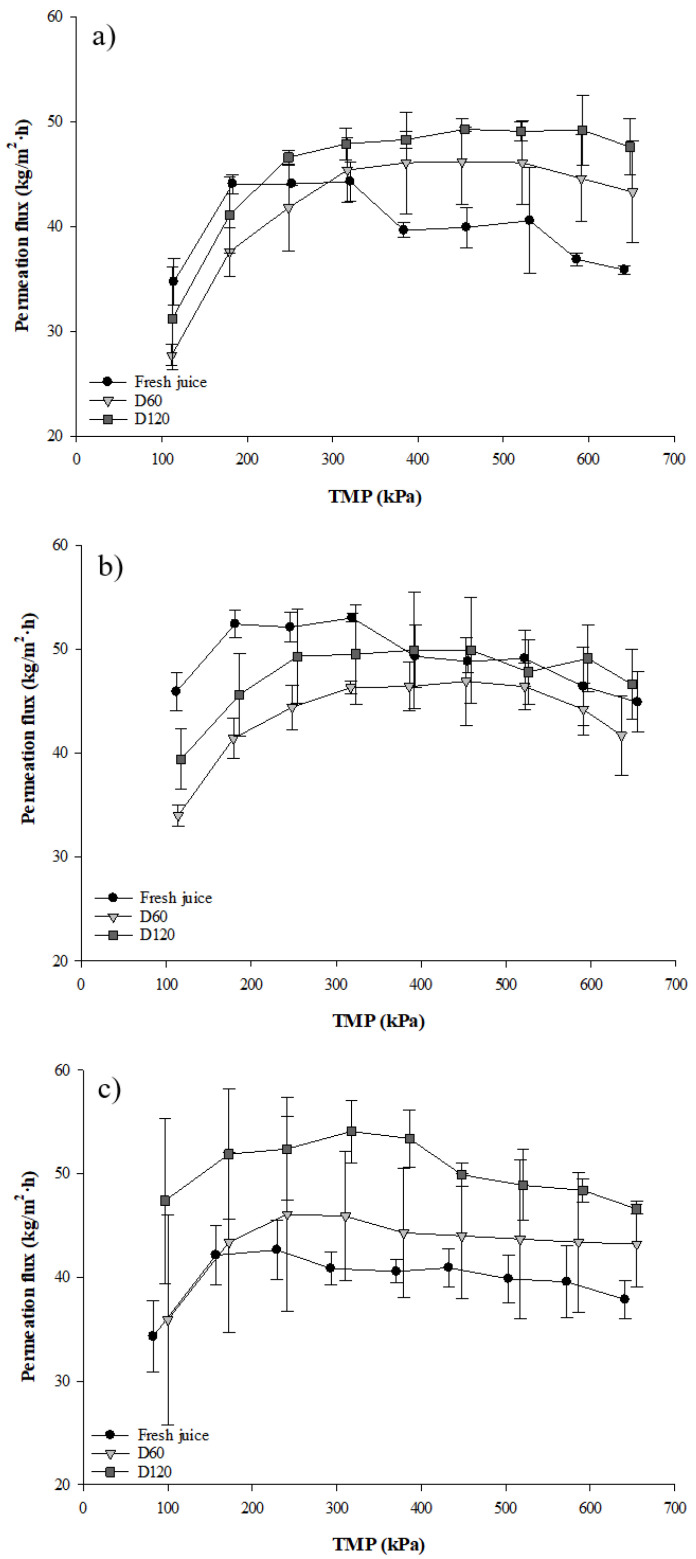
Permeation flux–transmembrane pressure (TMP) relationship for fresh and depectinized (D60 and D120) cranberry juices clarified by ultrafiltration (UF) with membrane MWCO of (**a**) 50 kDa, (**b**) 100 kDa and (**c**) 500 kDa.

Figure 1 presents the permeate flux-transmembrane pressure relationship in total recirculation mode for fresh and depectinized (D60 and D120) cranberry juices ultrafiltered with three different membrane MWCOs (50, 100 and 500 kDa). The pure water flux increased linearly with pressure from 152 to 682 L/m^2^.h for the 50 kDa UF membrane; 266 to 1246 L/m^2^.h for the 100 kDa membrane and 344 to 1583 L/m^2^.h for the 500 kDa membrane (Table A1). The permeate fluxes during UF of fresh and depectinized juices (Figure 1) were lower than pure water flux. The shapes of the curves were similar whatever the type of juice and membrane MWCO. At low TMP, the permeate flux was proportional to the TMP, and as the TMP increased, the permeate flux deviated from a linear flux-TMP behavior to decline with increasing TMP. The evolution of permeate flux differed as a function of the type of juices (fresh and depectinized) and the membrane MWCO. Indeed, during juice recirculation with a 50 kDa UF membrane (Figure 1a), the permeate fluxes of fresh juice were lower than depectinized juices above a TMP of 300 kDa. When a 100 kDa UF membrane was used, the depectinization treatment had no impact on permeate flux except at the very beginning of UF where permeate fluxes were higher for fresh juice than for depectinized juices. When a 500 kDa UF membrane was used, permeate fluxes calculated for the D120 juice were higher than for fresh juice (*p* < 0.05) and no difference was obtained between the D60 and D120 juices (*p* > 0.05).

The permeate flux–TMP relationship (Figure 1) allowed us to determine J_crit_ and J_lim_ parameters for fresh and depectinized juices clarified by UF with different membrane MWCO (Table 1). Critical flux is the lowest flux that creates irreversible fouling on a filtration membrane and was obtained when a deviation from linearity was observed in the TMP–flux relationship. Limiting flux is the highest flux obtained as a function of TMP applied. While similar J_lim_ values (average of 48.4 ± 4.8 kg/m^2^·h) were calculated for all types of juices and UF MWCO (*p* > 0.05), J_crit_ values differed as a function of MWCO and the type of juice (Table 1). However, no correlation was found between these parameters since a non-linearity was observed from the very beginning in the TMP–flux relationship. The optimal TMP to apply during concentration mode experiments for each condition (type of juice and membrane MWCO) corresponded to TMP obtained from Jcrit calculated for all conditions (type of juice and membrane MWCO) (Table 1). Globally, fresh juices had a lower optimal TMP compared to the D60 and D120 juices for all membrane MWCO. Similar optimal TMPs were obtained for both D60 and D120 juices for all membrane MWCO except the 100 kDa UF membrane (*p* < 0.05) (Figure 1).

### 3.2. Concentration Mode

**Figure 2:**
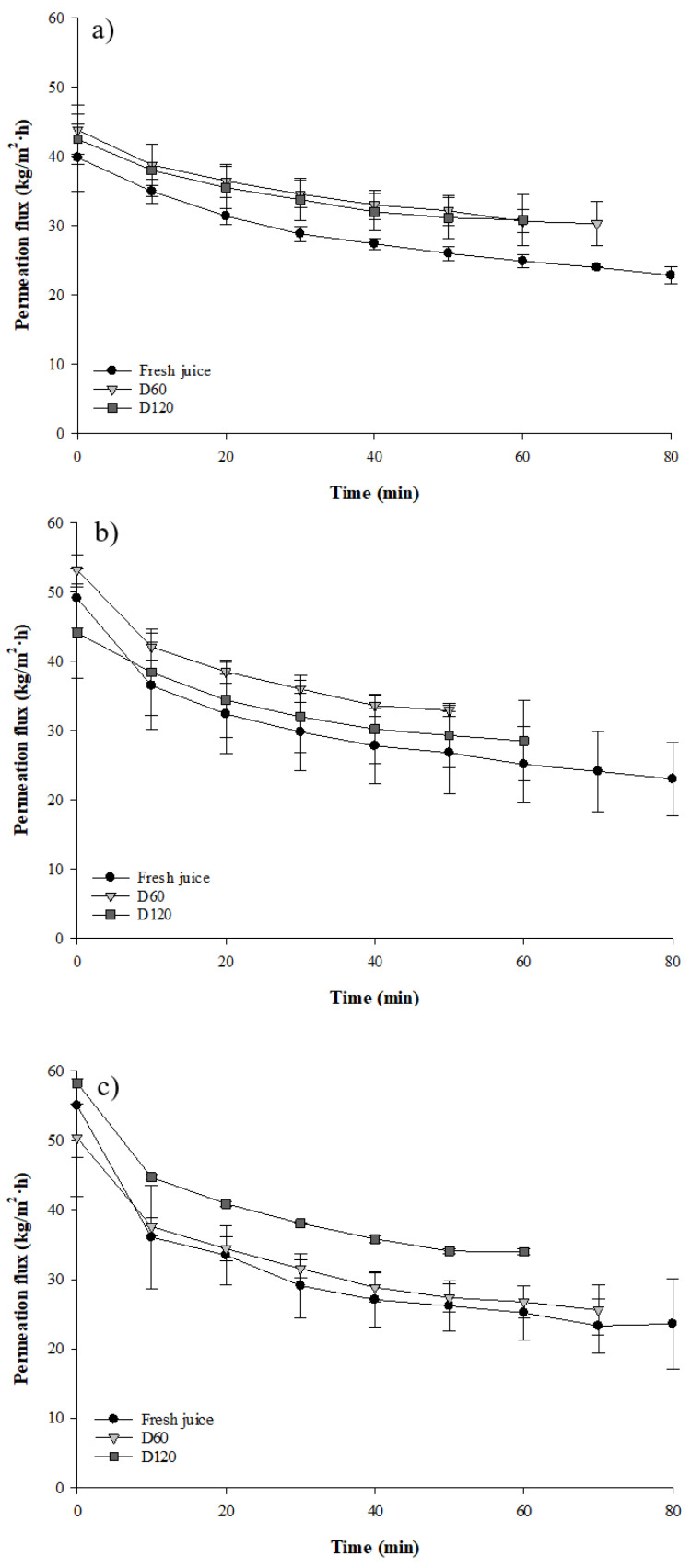
Evolution of permeation flux (kg/m^2^·h) of fresh and depectinized (D60 and D120) cranberry juices during clarification by UF with membrane MWCO of (**a**) 50 kDa, (**b**) 100 kDa and (**c**) 500 kDa membranes.

To evaluate the impact of the depectinization duration and membrane MWCO on UF performance during clarification of the cranberry juices, concentration mode was performed at the optimal TMP values obtained in total recycle mode (Table 1). For all juice types (fresh, D60 and D120), the initial permeate fluxes increased with the increase of membrane MWCO (Figure 2). More specifically, permeate flux increased by 29% between the fresh juice and D120 and by 11% between D60 and D120. Moreover, compared to the depectinized juices, the duration of UF for the fresh juice was 21% longer than D60 and 25% longer than D120, for all UF MWCO. The depectinization treatments (D60 and D120) did not impact the initial permeate flux compared to fresh juice, but it decreased the filtration duration. Additionally, whatever the MWCO, the total flux decline was higher for the fresh juice (42 to 57.6%) compared to depectinized juices at 60 min (29.4 to 45.2%) and 120 min (27.5 to 41.5%) (*p* < 0.05) (Table 2). Moreover, flux decline for cranberry juices (fresh, D60 and D120) clarified with the 50 kDa MWCO membrane was lower compared to the 100 and 500 kDa MWCO, except for D120, where similar flux declines for 50 and 100 kDa were calculated (*p* < 0.05). Finally, the duration of depectinization had no impact on total flux decline (*p* < 0.05).

### 3.3. Physicochemical Characteristics of Fresh, Depectinized and Clarified Cranberry Juices

The physicochemical characteristics of fresh, D60 and D120 juices before UF (non-clarified) and after UF (clarified) are presented in Table 3. Fresh and clarified juices had acidic pH values ranging from 2.66 to 2.87. Overall, clarified juices recovered after clarification by UF with membrane MWCO of 50 and 100 kDa had higher pH values than non-clarified juices and those recovered after UF with a membrane MWCO of 500 kDa. °Brix values were similar for all membrane MWCO for all types of juice (*p* > 0.05). However, non-clarified juices had higher °Brix compared to clarified juices whatever the depectinization conditions (*p* < 0.05). The titratable acidity was globally higher for non-clarified juices compared to the respective juices ultrafiltered at 50, 100 and 500 kDa. The clarity of non-clarified juices was systematically lower than clarified juices (*p* < 0.05). Clarified juices recovered after UF with membrane MWCO of 50 and 100 kDa had a higher clarity compared to those obtained with membrane MWCO of 500 kDa.

### 3.4. Polyphenol Content of Fresh, Depectinized and Clarified Cranberry

As observed in Table 3, the depectinization treatment had a significant effect on the concentration of total polyphenols in non-clarified juices (*p* < 0.05) since increases of 41 and 61% of polyphenol were obtained in the D60 and D120 juices, respectively, compared to fresh juices. The depectinization treatment and UF membrane MWCO did not greatly impact the total polyphenol content of cranberry juices. Except for the fresh juice ultrafiltered at 500 kDa, the total polyphenol content of clarified juices recovered after filtration with UF membrane were similar, regardless of the MWCO (*p* < 0.05). The content of total PAC ranged from 61 to 87 ppm whatever the type of juice and UF treatment applied for clarification. The depectinization treatment had no impact on total PAC concentrations in non-clarified juices. Similarly, total PAC content was the same for non-clarified and UF-clarified juices using membrane MWCO of 50, 100 and 500 kDa. Contrary to results obtained for total PACs, the depectinization treatment had a drastic impact on anthocyanin content (*p* < 0.05) since D60 and D120 juices lost 47 and 59% anthocyanin, respectively. However, the membrane MWCO used during clarification by UF had no impact on anthocyanin content (*p* > 0.05).

### 3.5. Characterization of Fouling Material

The characterization of the fouling material extracted from the surface of UF membranes used to clarify fresh and depectinized juices is presented in Table 4. No PACs were extracted for all types of cranberry juices and membrane MWCO (results not presented). Fouling by total polyphenols was influenced by depectinization treatment and the membrane MWCO (*p* < 0.05). Higher concentrations of total polyphenols were extracted from the 50 and 100 kDa MWCO UF membrane surfaces used to clarify D60 and D120 juices. Anthocyanin fouling at the UF membrane surface was mainly governed by the MWCO since anthocyanins extracted from the 50 and 100 kDa UF membranes were two to three times higher than that of the 500 kDa membrane.

## 4. Discussion

The purpose of this study was to evaluate UF performance during the clarification of fresh and depectinized cranberry juices using different UF membrane MWCO. These results provide the first evidence that both the MWCO and the type of juice impacted UF performance. Overall, depectinization treatment had a positive impact on permeate flux for low membrane MWCO. These effects were related to the composition of juice, the impact of depectinization on pectin hydrolysis and fouling by polyphenols.

### 4.1. Time Dependence between Permeate Flux and Transmembrane Pressure

In total recirculation experiments, only variations of TMP applied to the different cranberry juices negatively affected filtration performance. The evolution of permeate flux observed in Figure 1 had the typical shape of curves obtained during total recirculation experiments of other fruit juices such as kiwi and black currant juices [18,33,34]. Initially, at low TMP, more juice components (pectin, sugar, organic acid, etc.) passed through the membrane pores. However, at higher TMPs, the permeate flux plateaued due to a large accumulation of juice components in the polarization layer, which induced membrane fouling and increased the resistance to permeate [35]. More specifically, the results demonstrated that UF performance was mainly dependent on the type of juice (fresh and depectinized) since depectinization improved the permeate fluxes compared to fresh juices. The cell walls of fruits, including cranberry, consist mainly of pectin and cellulosic materials. Since pectin-rich fruit juice is generated after extraction from the fruit, the addition of pectinolytic enzymes before UF decreases the pectin content in depectinized juice, which improves the permeate flux [18]. In general, it is expected that the higher the MWCO of a membrane, the higher the permeate flux produced. While this tendency was observed for the 50 and 500 kDa-UF membranes, the evolution of permeate flux for the 100 kDa-UF membrane was similar to the 50 kDa UF membrane (Figure 1). This result was due to the very similar MWCO between the 50 and 100 kDa UF membranes, as explained by Echavarria, et al. [36] for filtration of apple juice with 100 kDa and 300 kDa UF membranes. Besides permeate flux, Jcrit and Jlim are good indicators of UF performance. No differences were observed between Jlim values for any of the treatments applied to the juice or for the different UF membrane MWCO. Consequently, the saturation of UF membrane by juice components appeared to be similar for fresh and depectinized juices [37]. However, even if no clear tendency was observed for the Jcrit values, different phenomena explain this parameter, such as adsorption of juice components at the surface or in the membrane, or formation of a cake at the membrane surface [35]. Calculating optimal TMP values for concentration mode experiments also helped explain the impact on system performance of treatments applied to cranberry juices (fresh and depectinized) and UF membrane MWCO. The lower optimal TMP for fresh juice compared to depectinized juice correlated with the early appearance of concentration polarization and the fouling phenomenon on the membrane surface due to the composition of fresh juice, with its higher pectin content [38].

### 4.2. Evolution of Permeate Flux during Clarification of Cranberry Juices

Experiments performed in concentration mode confirmed that the depectinization treatment improved the permeate flux and decreased the total flux decline. Moreover, the duration of the clarification step was reduced for depectinized juice compared to fresh juice. These results are consistent with previously published reports demonstrating that using pectinolytic enzymes improved the permeate fluxes of depectinized pomegranate [39] and apple juice [25,36]. Indeed, the decrease in UF performance during clarification of non-depectinized juices was mainly attributed to the retention of suspended compounds, causing reversible and irreversible fouling. Reversible fouling was explained mainly by the presence of pectin. However, in addition to pectin, polyphenolic compounds and proteins can combine to form soluble complexes that cause membrane fouling in the clarification of pomegranate juice [40]. The formation of a cake at the membrane surface, mainly caused by a fouling film composed of pectin, sugar and protein-polyphenol complexes, rapidly predominates and generates a drastic decrease in permeate fluxes, which explains the higher permeate flux decline calculated for fresh juices independent of the UF MWCO [41]. It is important to mention that the fouling was probably not induced by formation of a pectin gel since a UF temperature of over 50 °C would be needed to induce gelation [41,42]. Following the cake formation at the membrane surface, a smaller decrease in permeate flux was observed until the end of the filtration step, explained by a complete blockage of the pores by low molecular weight molecules (sugars and pectic residues), which compacted the membrane cake [39,43]. The global increase of permeate flux and decrease of total flux decline for depectinized juice were mainly due to pectin degradation by pectinolytic enzymes. Indeed, several authors have reported that hydrolysis of pectin into poly-*D*-galacturonic acid fragments reduced juice viscosity and the thickness of the cake layer, which improved UF performance [44]. We observed that the duration of depectinization treatment also had a positive impact on UF performance and reduced the time needed to reach the VCF of 3X. Lengthier depectinization treatments produced higher concentrations of low molecular weight oligomers (<2 kDa), resulting in higher permeate fluxes [25]. However, surprisingly, our results showed that the highest flux declines were calculated with the 100 and 500 kDa UF membranes. Girard and Fukumoto (2000) reported that the permeate flux might not increase with pore size since higher MWCO membranes can be more prone to fouling due to an internal pore blocking mechanism induced by penetration of particle, such as protein, polyphenol–protein complex and pectic substances into the pores [45,46].

### 4.3. Effect of Depectinization and Clarification on Physicochemical Characteristics and Suspended Solids of Fresh, Depectinized and Clarified Cranberry Juices

Our results showed that pH values were roughly similar for clarified fresh and depectinized juices (D60 and D120). However, the depectinization treatment decreased the pH of non-clarified cranberry juice (Table 3). This result agreed with the Baciu and Jördening [47] study on the kinetics of galacturonic acid release from sugar-beet pulp. These authors noticed that the use of a pectinase (Pectinex^®^) induced a pH reduction explained by the variation of galacturonic acid synthesis and probably by the action of esterases that liberated acetic acid in the juice [47]. Additionally, the increase of titratable acidity after depectinization is probably due to the release of galacturonic acid by the enzymatic degradation of pectin, mainly composed of poly-*D*-galacturonic acid units [48,49]. Similar values of clarity were obtained for clarified fresh and depectinized cranberry juices. The increase in clarity for clarified cranberry juices was consistent with similar studies performed on a wide range of fruit juices, including cherry juice [50], tangerine orange [51] juice and apple juice [38]. Indeed, clarification using UF eliminates pectic substances and colloidal particles concentrated in the UF retentate [52]. As expected, the combination of depectinization with UF improved the clarity of cranberry juice since the low molecular weight pectic residues generated from pectin hydrolysis pass through the UF membrane [25]. Finally, the lower °Brix values obtained for all clarified juices compared to non-clarified juices (Table 3) can be explained by the removal of suspended solids, including pectin residues that remained in the UF retentate [45].

### 4.4. Effect of Depectinization and Clarification on Polyphenol Content of Fresh, Depectinized and Clarified Cranberry Juices

PACs are polymeric chains of flavonols mainly composed of epicatechin or catechin subunits. In cranberry juice, low molecular weight PACS were mostly present in their 1–2 mers forms followed by monomers and 4–6 mers [53]. The non-significant impact of clarification by UF on PACS content could be related to their low molecular weight compared to membrane MWCO, which could explain their absence in the fouling layer. As shown in Table 3, and contrary to previous reports [54,55,56], UF of cranberry juices had no impact on anthocyanin loss. However, anthocyanins were affected by the depectinization treatment since their concentrations were drastically reduced in depectinized juices compared to fresh juices. During juice treatment by pectinolytic enzymes and clarification, different factors are known to affect the stability of anthocyanins. Anthocyanins are affected by heat treatments following a first-order kinetic model [57,58,59]. Patras et al. [60] reported that anthocyanins from strawberry and blackberry purées were impacted by heat treatment at 70 °C for 2 min, compared to untreated purées. In addition, when heated at 90 °C prior to depectinization with pectinolytic enzymes, red raspberry juice was negatively affected by depectinization treatment [61]. Consequently, it is highly probable that the heat treatment applied in this study for pectinolytic enzyme inactivation (80 °C for 2 min) negatively affected anthocyanin content. Furthermore, other studies have demonstrated that commercial pectinolytic enzyme reduced the total content of anthocyanins in raspberry [62] and strawberry [63,64] juices due to the presence of β-glycosidase activity present in the enzyme preparation [65]. Finally, a small amount of anthocyanin loss was related to fouling at the surface of the UF membrane, mainly for 50 and 100 kDa MWCO (Table 4). To the best of our knowledge, no available study describes the binding mechanism between UF material and anthocyanin. However, it was observed that interaction between anthocyanin, pectin and pectic residues occurred in blueberry model juice and this interaction was improved at low pH [66]. Consequently, the presence of anthocyanin as a fouling component could be explained by the interaction between anthocyanin and pectin/pectic substances adsorbed at the surface of the UF membrane. Similar to anthocyanins, total polyphenols were affected by the depectinization treatment, but were also affected by the clarification process, independent of the MWCO. As explained for anthocyanins, heat treatment applied to cranberry juices (80 °C for 2 min) could decrease the polyphenol content. Indeed, as demonstrated in strawberry juices, a 60 s heat treatment at 90 °C decreased the polyphenol content compared to unprocessed juice [67]. The loss of total polyphenols was correlated with their recovery at the UF membrane surface. Indeed, polyphenols were recognized as fouling material [68,69] at the membrane surface by adsorptive mechanisms via hydrogen bonding and hydrophobic interactions [70]. The effect of pore size on adsorptive polyphenol fouling is particularly interesting since the 500 kDa UF was less subject to fouling than the 50 and 100 kDa UF membranes. As published by Susanto et al. (2009), membranes with larger pore sizes resulted in less fouling by polyphenol than membranes with smaller pore sizes [70].

## 5. Conclusions

Depectinization treatment reduced the duration of clarification by UF and improved the permeate fluxes. Contrary to PACS, total polyphenol and anthocyanins were negatively affected by the depectinization and UF treatments, probably due to the heat treatment to inactivate pectinolytic enzymes. Related to their bioactive properties, the total loss of polyphenol and anthocyanins in clarified and depectinized juices could potentially affect the nutritional quality of juice. Membrane fouling by these two components led to the reduction of their content in clarified juice despite the fact that using a high membrane MWCO limited their presence in the fouling layer. Overall, a 60 min depectinization coupled to clarification by a 500 kDa UF membrane represents a good compromise between the enhancement of filtration performance and the loss of polyphenols and their fouling at the membrane surface. This process combination, which must be validated during juice production at the industrial-scale, is of major importance for the cranberry industry with potential improvement in juice productivity and quality.

## Figures and Tables

**Table 1:** Values of optimal TMP, critical flux (Jcrit) and limiting flux (Jlim) during total recirculation of raw and depectinized (D60 and D120) cranberry juices clarified by UF with membrane MWCO of 50, 100 and 500 kDa.

	UF Membrane MWCO (kDa)	J_crit_ (kg/m^2^·h)	J_lim_ (kg/m^2^·h)	Optimal TMP (kPa)
**Fresh juice**	**50**	44.1 ± 0.9 ^a,b^	44.8 ± 1.1 ^a^	192 ± 2.9 ^b^
	**100**	52.5 ± 0.7 ^c^	53.2 ± 0.1 ^a^	191 ± 4.0 ^b^
	**500**	42.1 ± 3.8 ^a^	42.6 ± 3.1 ^a^	153 ± 1.2 ^a^
**D60**	**50**	42.9 ± 2.6 ^a^	46.5 ± 4.5 ^a^	241 ± 3.4 ^d^
	**100**	43.2 ± 1.9 ^a^	47.9 ± 2.8 ^a^	205 ± 2.2 ^c^
	**500**	46.5 ± 6.3 ^a,b^	47.1 ± 8.0 ^a^	241 ± 4.7 ^d^
**D120**	**50**	46.6 ± 0.6 ^b^	50.4 ± 1.8 ^a^	243 ± 0.9 ^d^
	**100**	47.0 ± 4.2 ^a,b,c^	51.0 ± 5.4 ^a^	205 ± 8.0 ^c^
	**500**	52.2 ± 2.4 ^c^	54.2 ± 3.0 ^a^	252 ± 10 ^d^

Data with different letters (a–c) in the same column are significantly different (LSD, *p* < 0.05). A two-way analysis of variance (ANOVA) was performed at a 95% confidence level. Fisher’s protected least significant difference (LSD) was used to compare membrane cut-offs and juice depectinization treatments.

**Table 2:** Total flux decline (%) of fresh and depectinized (D60 and D120) cranberry juices after UF with membrane MWCO of 50, 100 and 500 kDa.

	Total Flux Decline (%)		
	Fresh Juice	D60	D120
**50 kDa**	42.0 ± 5.5 ^c^	29.4 ± 4.1 ^d^	27.5 ± 3.3 ^d^
**100 kDa**	55.3 ± 9.7 ^a,b^	38.4 ± 3.2 ^c^	35.7 ± 4.7 ^c,d^
**500 kDa**	57.6 ± 5.9 ^a^	45.2 ± 14.3 ^b,c^	41.5 ± 2.6 ^c^

Data with different letters (a–d) in the same column are significantly different (LSD, *p* < 0.05). A two-way analysis of variance (ANOVA) was performed at a 95% confidence level. Fisher’s protected least significant difference (LSD) was used to compare membrane cut-offs and juice depectinization treatments.

**Table 3:** Physicochemical characteristics and polyphenol concentrations of fresh and depectinized (D60 and D120) cranberry juices before UF (control), as well as their respective permeates generated after UF with membrane molecular weight cut-off (MWCO) of 50, 100 and 500 kDa.

		pH	°Brix	Titratable Acidity (g/L Citric Acid Equivalent)	Clarity (%T)	Total Polyphenols (mg/L)	Total PACs (ppm)	Total Anthocyanins (ppm)
**Fresh juice**	**Non-clarified**	2.77 ± 0.02 ^b,c^	4.0 ± 0.2 ^b,c^	13.0 ± 0.14 ^b,c^	40.9 ± 1.0 ^f^	358 ± 53 ^d^	78 ± 10 ^a,b,c,d,e^	68 ± 8 ^a^
	**50 kDa**	2.81 ± 0.01 ^a,b^	3.7 ± 0.1 ^d,e,f^	11.8 ± 0.3 ^e,f^	95.3 ± 1.8 ^a,b^	495 ± 23 ^b^	66 ± 6 ^e,f^	71 ± 3 ^a^
	**100 kDa**	2.85 ± 0.02 ^a^	3.5 ± 0.1 ^e,f^	11.7 ± 0.5 ^f^	94.0 ± 1.2 ^a,b^	473 ± 15 ^b^	61 ± 10 ^e,f^	70 ± 3 ^a^
	**500 kDa**	2.77 ± 0.02 ^b,c^	3.5 ± 0.1 ^f^	11.9 ± 0.5 ^f^	78.0 ± 2.9 ^e^	360 ± 62 ^d^	70 ± 10 ^d,e,f^	59 ± 12 ^b^
**D60**	**Non-clarified**	2.70 ± 0.03 ^d,e^	4.4 ± 0.3 ^a^	13.2 ± 0.02 ^b^	36.7 ± 0.2 ^g^	506 ± 21 ^b^	80 ± 3 ^a,b,c,d^	38 ± 4 ^c,d^
	**50 kDa**	2.85 ± 0.01 ^a^	3.7 ± 0.0 ^d,e^	12.0 ± 0.2 ^e,f^	95.1 ± 2.5 ^a,b^	465 ± 5 ^b,c^	78 ± 8 ^a,b,c,d,e^	39 ± 1 ^c^
	**100 kDa**	2.86 ± 0.02 ^a^	3.7 ± 0.1 ^d,e^	12.3 ± 0.2 ^d,e^	97.4 ± 2.5 ^a^	463 ± 28 ^b,c^	73 ± 2 ^c,d,e,f^	35 ± 2 ^c,d,e^
	**500 kDa**	2.75 ± 0.06 ^c,d^	3.8 ± 0.1 ^c,d^	12.7 ± 0.7 ^b,c,d^	80.3 ± 1.8 ^e^	397 ± 88 ^c,d^	72 ± 16 ^c,d,e,f^	38 ± 9 ^c,d^
**D120**	**Non-clarified**	2.66 ± 0.02 ^e^	4.1 ± 0.0 ^b^	13.8 ± 0.02 ^a^	25.6 ± 0.1 ^h^	578 ± 33 ^a^	91 ± 3 ^a^	31 ± 4 ^c,d,e^
	**50 kDa**	2.83 ± 0.01 ^a^	3.8 ± 0.1 ^c,d^	12.6 ± 0.3 ^c,d^	90.1 ± 0.5 ^c^	462 ± 50 ^b,c^	83 ± 6 ^a,b,c^	29 ± 1 ^d,e^
	**100 kDa**	2.87 ± 0.01 ^a^	3.8 ± 0.1 ^c,d^	12.3 ± 0.1 ^d,e^	91.7 ± 1.8 ^b,c^	450 ± 16 ^b,c^	76 ± 4 ^b,c,d,e^	28 ± 1 ^e^
	**500 kDa**	2.70 ± 0.08 ^d,e^	3.8 ± 0.1 ^c,d^	12.9 ± 0.5 ^b,c^	86.0 ± 3.3 ^d^	498 ± 16 ^b^	87 ± 2 ^a,b^	27 ± 6 ^e^

Data with different letters (a–g) in the same column are significantly different (LSD, *p* < 0.05). A two-way analysis of variance (ANOVA) was performed at a 95% confidence level. Fisher’s protected least significant difference (LSD) was used to compare membrane cut-offs and juice depectinization treatments.

**Table 4:** Characterization of fouling material extracted from UF membrane surfaces after clarification of fresh and depectinized (D60 and D120) cranberry juices.

		Total Polyphenols (mg/L)	Total Anthocyanins (ppm)
**Fresh juice**	**50 kDa**	232 ± 49 ^c,d,e^	22 ± 7 ^b^
	**100 kDa**	259 ± 28 ^b,c,d^	25 ± 8 ^a,b^
	**500 kDa**	102 ± 34 ^e^	12 ± 5 ^c^
**D60**	**50 kDa**	354 ± 89 ^a,b^	28 ± 5 ^a,b^
	**100 kDa**	360 ± 42 ^a,b^	27 ± 7 ^a,b^
	**500 kDa**	123 ± 55 ^e^	9 ± 4 ^c^
**D120**	**50 kDa**	310 ± 113 ^b,c^	23 ± 2 ^a,b^
	**100 kDa**	460 ± 98 ^a^	33 ± 8 ^a^
	**500 kDa**	182 ± 19 ^d,e^	10 ± 1 ^c^

Data with different letters (a–e) in the same column are significantly different (LSD, *p* < 0.05). A two-way analysis of variance (ANOVA) was performed at a 95% confidence level. Fisher’s protected least significant difference (LSD) was used to compare membrane cut-offs and juice depectinization treatments.

## Data Availability

Data is contained within the article.

## Appendix A

**Table A1:** Pure water fluxes of 50, 100 and 500 kDa UF membranes measured for transmembrane pressure ranging from 15 to 95 kPa.

	Water Flux (L/m^2^·h)		
	50 kDa	100 kDa	500 kDa
**Fresh juice**	164 ± 9 to 792 ± 68	266 ± 8 to 1169 ± 29	344 ± 83 to 890 ± 86
**D60**	173 ± 22 to 773 ± 46	ND	374 ± 0 to 910 ± 0
**D120**	152 ± 26 to 682 ± 26	299 ± 24 to 1246 ± 126	587 ± 111 to 1583 ± 81

ND: not determined.
